# Trials using deferred consent in the emergency setting: a systematic review and narrative synthesis of stakeholders’ attitudes

**DOI:** 10.1186/s13063-022-06304-x

**Published:** 2022-05-16

**Authors:** Aran Fitzpatrick, Fiona Wood, Victoria Shepherd

**Affiliations:** 1 School of Medicine, Cardiff University, Cardiff, Wales; 2 Division of Population Medicine and PRIME Centre Wales, University Hospital of Wales, Cardiff University, 8th floor Neuadd Meirionnydd, Heath Park, Cardiff, CF14 4YS Wales; 3 Centre for Trials Research, Cardiff University, Cardiff, Wales

**Keywords:** Critical care, ICU, ITU, Intensive care, Recruitment method, Recruitment strategy, Systematic review

## Abstract

**Background:**

Patients with acute conditions often lack the capacity to provide informed consent, and narrow therapeutic windows mean there is no time to seek consent from surrogates prior to treatment being commenced. One method to enable the inclusion of this study population in emergency research is through recruitment without prior consent, often known as ‘deferred consent’. However, empirical studies have shown a large disparity in stakeholders’ opinions regarding this enrolment method. This systematic review aimed to understand different stakeholder groups’ attitudes to deferred consent, particularly in relation to the context in which deferred consent might occur.

**Methods:**

Databases including MEDLINE, EMCare, PsychINFO, Scopus, and HMIC were searched from 1996 to January 2021. Eligible studies focussed on deferred consent processes for adults only, in the English language, and reported empirical primary research. Studies of all designs were included. Relevant data were extracted and thematically coded using a narrative approach to ‘tell a story’ of the findings.

**Results:**

Twenty-seven studies were included in the narrative synthesis. The majority examined patient views (*n* = 19). Data from the members of the public (*n* = 5) and health care professionals (*n* =5) were also reported. Four overarching themes were identified: level of acceptability of deferred consent, research-related factors influencing acceptability, personal characteristics influencing views on deferred consent, and data use after refusal of consent or participant death.

**Conclusions:**

This review indicates that the use of deferred consent would be most acceptable to stakeholders during low-risk emergency research with a narrow therapeutic window and where there is potential for patients to benefit from their inclusion. While the use of narrative synthesis allowed assessment of the included studies, heterogeneous outcome measures meant that variations in study results could not be reliably attributed to the different trial characteristics. Future research should aim to develop guidance for research ethics committees when reviewing trials using deferred consent in emergency research and investigate more fully the views of healthcare professionals which to date have been explored less than patients and members of the public.

Trial registration

PROSPERO CRD42020223623

**Supplementary Information:**

The online version contains supplementary material available at 10.1186/s13063-022-06304-x.

## Background

Medical research is essential as treatments and interventions should be proven to be effective before their implementation in healthcare. Informed consent is a pivotal part of research ethics in order to protect the autonomy and right to self-determination of participants [1]. In addition, the ethical principle of justice argues that patients who are unable to provide consent for themselves should be given the opportunity to participate in research. There is also growing recognition of the importance of research conducted on populations who lack the capacity to consent for themselves (either permanently or temporarily) as the alternative is to continue to use unproven interventions in these most vulnerable groups of patients [2]. For informed consent to be valid, a participant must have the capacity, that is, they must be able to understand information given to them, retain and weigh up the necessary information, and communicate their decision [3].

Despite its importance, informed consent is not always feasible in emergency research. Patients with acute conditions such as seizures, sepsis, and traumatic brain injuries require time-critical care and often lack the capacity to provide informed consent [4]. The narrow therapeutic window means that there is no time to seek consent from a surrogate [5]. A UK trial found only 2.6% of research subjects in an intensive care unit trial analysing the use of pulmonary artery catheters could provide informed consent before randomisation [6]. This inability to obtain consent prospectively raises a number of practical and ethical issues around how best to recruit participants to research in emergency settings.

In 2013, the World Medical Association outlined the criteria to permit ‘research without prior consent’ (RWPC) in emergency settings [1]. The criteria stated that if informed consent cannot be obtained from an incapacitated patient in the time frame of the patient’s condition, and specific criteria included in the research ethics committee-approved study protocol are met, informed consent can be deferred. Consent from either the participant or a legal representative must then be obtained as soon as possible after enrolment in the study, a process known as deferred consent. If deferred consent is given, that participant is able to continue in the trial and permission has been given for researchers to use data that has already been collected in their analysis as well as any continued data. The use of the term deferred consent has received some criticism due to the implication that consent is just delayed, with some preference for the alternative term RWPC [7]. However, in this paper, the authors have chosen to continue to use the term deferred consent as it continues to be widely used in practice [4, 8]. During the COVID-19 pandemic, alternative consent models such as deferred consent have been used to enrol critically ill patients into vital emergency research testing the efficacy of therapeutics to combat the disease. For example, deferred consent has been successfully used in the REMAP-CAP trial, an international adaptive platform trial testing multiple therapies for COVID-19 [9].

There are international differences between the legal frameworks governing research in emergency settings. In the USA, RWPC is permitted under the Exception From Informed Consent code of Federal Regulations [10]. This regulation allows for the patient to continue in the study even if they do not give their consent once consciousness is regained. However, some states have imposed more restrictive requirements. A key requirement of research under the EFIC pathway is that investigators must disseminate information about their research and solicit feedback from community stakeholders. There are similar disparities amongst the European Union (EU) member states with approximately half legally permitting deferred consent [11, 12]. RWPC is also permissible in Canada and parts of Australasia and the UK through both the 2005 Mental Capacity Act [3] and the 2006 Amendment to the 2004 EU Clinical Trials Regulations [13].

Despite its legal standing, there are still debates over whether deferred consent is ethical, amid concerns that it fails to respect the individual’s autonomy [13, 14]. This includes ‘borderline’ situations where the urgency of treatment and the patient’s (in)ability to provide prospective consent are less explicit. Many empirical studies have explored the views of relevant groups involved in the RWPC process. However, they report conflicting stakeholder views [15–17]. These uncertainties make the application of the regulatory frameworks difficult, can lead to recruiting fewer participants, and result in a lack of effective treatment in emergency settings. Understanding key stakeholders’ views regarding deferred consent would enable researchers to design and conduct emergency research in a way that is most acceptable to all stakeholder groups. To date, there is no single review synthesising the attitudes of different stakeholders regarding the use of deferred consent in emergency settings.

This systematic review aims to synthesise existing studies to understand the attitudes of key stakeholders (including healthcare professionals (HCPs), researchers, patients, and members of the public) towards the use of deferred consent in emergency research settings, particularly in relation to the context in which it might occur.

## Methods

A systematic review methodology was used [18]. The review is reported according to the Preferred Reporting Items for Systematic Reviews and Meta-Analysis guidelines (PRISMA Checklist; Additional file 1). The protocol was prospectively registered in the PROSPERO database (CRD42020223623). A narrative synthesis, synthesising qualitative and quantitative data to ‘tell a story’ of the results, was performed in line with the Cochrane guidance [19].

### Eligibility criteria

The search was limited to papers published since 1996 in the English language. The cut-off date was chosen due to the publication of the International Conference on Harmonisation Good Clinical Practice Guidelines in 1996 which set standards for the performance of clinical trials to protect the rights, safety, and well-being of research participants including how to manage consent [20]. The search was limited to adults only as consent processes for paediatric research are very different and have been explored in previous research [21]. Studies of all designs were included that reported empirical primary research, utilising either qualitative and/or quantitative methods. The full inclusion and exclusion criteria are listed in Table 1. Of note, we did include EFIC studies but only if they stated that participants were later informed about their participation and their consent was sought to remain in the study.

**Table 1:** Inclusion and exclusion criteria

Inclusion	Exclusion
Studies that report views of key stakeholders of deferred consent (HCPs, researchers, patients, family members, members of the public)	Research not appropriate for deferred consent (elective research, standard clinical procedures, vaccinations, screening)
Studies focusing on the procedure of deferred consent in research	Studies not reporting empirical research data (opinion pieces, descriptive processes, editorials)
Empirical research, using qualitative and/or quantitative methods, on gathering data on views of deferred consent from key stakeholders	Unpublished dissertations, conference abstracts, reports, protocol papers
	Papers published before 1996
	Papers not in the English language
	Studies involving participants < 18 years old only

### Systematic search

Five electronic databases were searched (MEDLINE, EmCare, PsychINFO, Scopus, HMIC) for papers published from 1996 to the date the search was conducted (January 2021). The reference lists of key relevant papers were also searched. The search strategy, developed with input from a subject librarian, used four key concepts: key stakeholders, attitudes, consent methods, and emergency research. The MEDLINE search strategy is reported in Additional file 2. The results were imported into EndNote X9 and deduplicated, and title and abstract screening was performed. To ensure that the inclusion and exclusion criteria were being met, 10% of results were double-screened independently by another member of the review team. Papers meeting the inclusion criteria were then exported into the Rayyan systematic review software for full-text assessment by two team members [22]. The papers were independently reviewed in line with the eligibility criteria, and reasons for exclusion were recorded. Inconsistencies between the results were discussed among the authors until a consensus was achieved. If the two authors could not agree, then a third member of the team would arbitrate the discussion. This was not required.

### Critical appraisal

The studies were critically appraised by one researcher using the Mixed Methods Appraisal Tool (MMAT) as it is designed to appraise a range of studies designs including mixed designs [23]. It includes five core quality criteria for each of the different types of study designs. The purpose of the quality assessment is to provide an assessment of the strength of the evidence available on which conclusions will be drawn. In accordance with the MMAT guidance, the overall scores for each study were not calculated, but the rating of each criterion was presented [23]. In line with the established approaches to conducting narrative syntheses, no studies were excluded based on their methodological quality [19]. Issues in the study design were noted and incorporated into the analysis of results.

### Data extraction

Data were extracted and inputted into a purposefully designed form (Additional file 3). Following the piloting of the tool, data extraction was performed, with 10% independently extracted by another team member. Data were imported into the NVivo 12 software for coding.

### Data synthesis

A narrative synthesis was performed in line with guidance proposed by Popay et al. [19]. This was an iterative process conducted over separate stages. A preliminary synthesis of findings was performed. Extracted study data were coded and organised into overarching themes. The relationships between the extracted data were then analysed and refined according to the characteristics of the study design, resulting in a synthesis of the included data.

## Results

### Systematic search

**Fig. 1:**
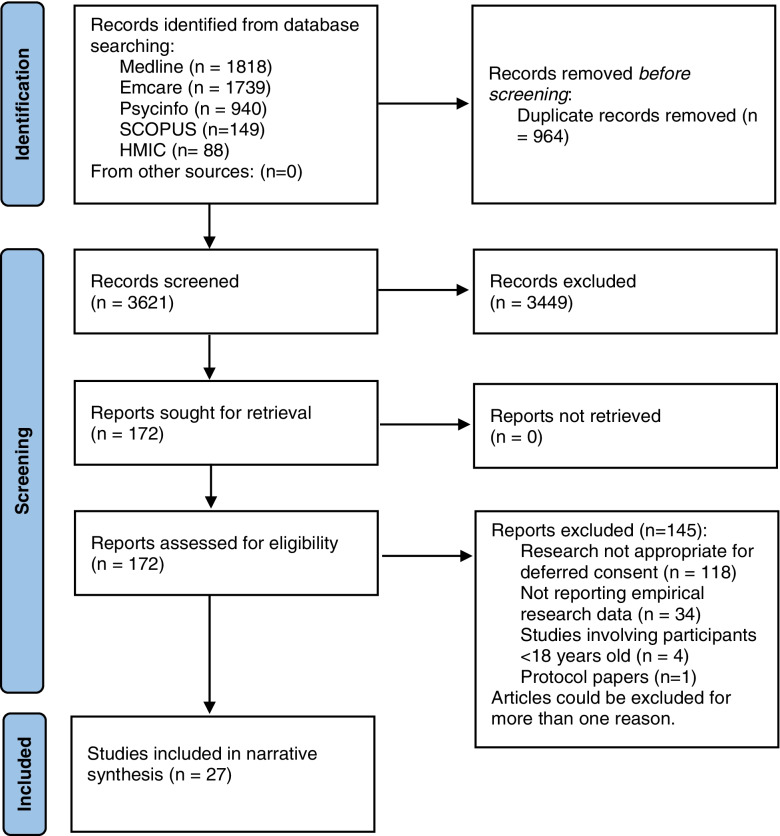
PRISMA flow diagram

Database searches returned 4734 potentially eligible papers with no additional papers identified through other sources, resulting in 3621 after deduplication. Of these papers, 3449 were excluded during the title and abstract screening leaving 172 papers for full-text assessment. Twenty-seven papers were included in the analysis. Search and screening details are recorded in the PRISMA flow diagram (Fig. 1).

The majority (*n* = 22) of studies investigated stakeholder views in the context of an intensive care unit (ICU)/hospital setting while four were situated in pre-hospital settings and one in obstetrics. Most studies examined patient views (*n* = 19). However, data from the members of the public (*n* = 5) and HCPs (*n* = 5) were also reported with some papers reporting views from more than one stakeholder group. Twenty-two studies were of quantitative design while five used qualitative methods. All except three papers were published between 2010 and 2020. The study characteristics are reported in Table 2.

**Table 2:** Table of Included studies

Study author and year	Country	Clinical context	Study design	Study aims	Scenario: real or hypothetical	Participant characteristics	Quality appraisal
Armstrong, S. et al. (2019) [24]	UK	Ambulance trials	Qualitative study	Understand the views of and experiences of expert informants	Real experiences and views of researchers regarding pre-hospital ambulance research	Academic researchers: *n* = 11 Clinical researchers: *n* = 3	High
Beshansky, J. R. et al. (2014) [25]	USA	Acute myocardial infarction	Quantitative descriptive study	Evaluate the utility of telephone survey data done as part of the EFIC process	As part of the IMMEDIATE trial investigating pharmacological myocardial metabolic support for acute myocardial infarction	Community members surveyed: *n* = 2079 Patients eligible for the study: *n* = 828	High
Booth, M. G. et al. (2005) [26]	UK	Cardiac arrest and severe trauma	Quantitative descriptive study	Assess public perception and attitudes	Two hypothetical scenarios of ICU research	Patients: *n* = 361	Low
Brown, P. et al. (2020) [27]	UK	Emergency department research	Qualitative descriptive study	Explore the experience of research nurses	Real experiences of healthcare professionals in ICU and emergency unit research	Research nurses: *n* = 10	High
Buckley, J. M. et al. (2016) [28]	UK	Emergency department research	Qualitative study	Investigate patients’ feelings	Hypothetical scenario of emergency research	Inpatients: *n* = 17	High
Burns, K. E. A. et al. (2011) [29]	Canada	Emergency department research	Quantitative descriptive study	Assess the general public’s attitudes	Three hypothetical scenarios of emergency research	Members of the public: *n* = 221	Low
Campwala, I. et al. (2020) [30]	USA	Haemorrhagic shock	Quantitative descriptive study	Learn about the experience of patients and surrogates and their general opinions	As part of the PAMPer study investigating pre-hospital plasma for haemorrhagic shock	Respondents in total: *n* = 93	Moderate
Cook, D. J. et al. (2008) [31]	Canada Australia NZ	Emergency department research	Quantitative descriptive study	Understand the experiences, beliefs, and practices of professionals involved in critical care research	Experiences of healthcare professionals and researchers in ICU and emergency unit research	Respondents (physicians, research coordinators, or others): *n* = 284	High
de Tonnerre, E. J. et al. (2020) [32]	Australia	Emergency department research	Quantitative descriptive study	Determine patients' perceptions	Real experiences and views of patients in emergency research	Suitable patients participated: *n* = 315/360	Moderate
Dickert, N. W. et al. (2017) [33]	USA	Acute myocardial infarction	Quantitative descriptive study	Explore views of patients	Hypothetical acute myocardial infarction study types	Patients with AMI: *n* = 30	Moderate
Dickert, N. W. et al. (2019) [34]	USA	Acute myocardial infarction and stroke	Quantitative descriptive study	Study experiences of patients	Real experiences of ICU patients in previous acute myocardial infarction/stroke research	Respondents by referred patients: *n* = 176/540 Patients: *n* = 107 Patient surrogates: *n* = 69	Moderate
Do Amaral Pfeilsticker, F. J. et al. (2020) [35]	Brazil	ICU research	Quantitative descriptive study	Address the willingness of patients to be enrolled in a scientific study as volunteers	Hypothetical ICU study	Pairs of ICU patients and their respective legal representatives: *n* = 208	Low
Furyk, J. et al. (2018) [21]	Australia	Emergency department research	Quantitative descriptive study	Give voice to the general public’s views of prospective and retrospective (deferred) consent	Hypothetical emergency research	Public responses: *n* = 1217	Moderate
Gigon, F. et al. (2013) [36]	Switzerland	ICU research	Quantitative descriptive study	Investigate the preferences of both patients and relatives	Two hypothetical ICU studies	Eligible patient responses: *n* = 185/472 Patient accompanied with relative: *n* = 125 Patient unaccompanied: *n* = 60	Moderate
Gobat, N. et al. (2019) [37]	Belgium, Spain, Poland, Ireland, the UK, Canada, Australia, New Zealand	Emergency department research	Quantitative descriptive study	Understand public views	Hypothetical scenarios of emergency research during an influenza pandemic	Members of the public: *n* = 6804	Moderate
Honarmand et al. (2018) [38]	Canada	ICU research	Prospective observational study	Describe the feasibility of the deferred consent model in a low-risk study	As part of the PRO-TROPICS trials	Critically ill patients in the ICU: *n* = 266/280	High
Houghton, G. et al. (2018) [39]	UK	Post-partum haemorrhage	Qualitative study	Investigate participants’ views	Clinical trial investigating the effect of tranexamic acid versus placebo for post-partum haemorrhage	Participants: *n* = 15 Consented while PPH was ongoing: *n* = 8 Consent was waived: *n* = 7	High
Kamarainen, A. et al. (2012) [40]	Finland	Cardiac arrest	Quantitative descriptive study	Survey the attitudes and experiences of surviving cardiac arrest victims, legal representatives, consent providers, and emergency physicians	As part of the pre-hospital index study investigating therapeutic hypothermia after cardiac arrest	Patients: *n* = 11/12 Consent providers: *n* = 17/25 Physicians: *n* = 13/13	High
Kleindorfer, D. et al. (2011) [41]	USA	Stroke	Quantitative descriptive study	Explore stroke survivors’ opinions	Hypothetical scenarios of stroke research	Ischaemic stroke patients (or proxies): *n* = 194/329	Moderate
Manda-Taylor, L. et al. (2019) [42]	Malawi	Emergency department research	Qualitative study	Examine the acceptability of deferred consent for research studies	Real experiences and views towards emergency department research	REC (research ethics committee) members: *n* = 5 Health care providers: *n* = 6 Local community members (one focus group): *n* = 12	High
Perner, A. et al. (2010) [43]	Denmark	ICU	Quantitative descriptive study	Survey attitudes amongst relatives of unconscious adult patients	Hypothetical ICU drug trial	Relatives of unconscious ICU patients: *n* = 42	High
Potter, J. E. et al. (2013) [44]	Australia	ICU	Quantitative descriptive study	Determine the opinion of participants	As part of the NICE-SUGAR study comparing blood glucose targets in ICU patients	Participants of the NICE-SUGAR study: *n* = 210	High
Scales, D. C. et al. (2009) [45]	Canada	ICU	Quantitative descriptive study	Determine patients’ preferences for different consent frameworks	Hypothetical study—randomised placebo-controlled trial of low-risk	Capable and consenting survivors of critical illness: *n* = 240	Moderate
Scicluna, V. M. et al. (2019) [46]	USA	AMI and stroke	Qualitative study	Explore the experiences of participants	Real experiences and attitudes of ICU patients towards their involvement in trials	Interviews: *n* = 27 Acute MI patients: *n* = 12 Stroke patients: *n* = 2 Surrogates for stroke patients: *n* = 13	High
Scicluna, V. M. et al. (2020) [47]	USA	Emergency department research	Quantitative descriptive study	Explore attitudes in patients	As part of the ESETT trial—comparing anticonvulsant therapies in status epilepticus	Participants: *n* = 317 Adult patients: *n* = 48 Surrogates for paediatric patients: *n* = 151 Surrogates for adult patients: *n* = 118	Moderate
Shamy, M. C. F. et al. (2019) [48]	Canada and Europe	ICU	Quantitative descriptive study	Investigate the knowledge and opinions of patients	As part of the ESCAPE trial—evaluating standard care plus thrombectomy in acute ischaemic stroke	Patients/authorised third parties who completed baseline survey: *n* = 33/56 Patients/authorised third parties who completed the 90-day follow-up survey: *n* = 27/56	Moderate
Terry, M. A. et al. (2017) [49]	USA	ICU	Prospective cohort study	Determine the extent to which ICU patients or surrogates support a deferred consent process for a minimal-risk study	As part of the microbiome study with added hypothetical scenarios	ICU patients: *n* = 135/157	Moderate

### Quality appraisal

The quality appraisal of included studies is reported in Table 2. Most studies were judged to be of high (*n* = 12) and moderate (*n* = 12) quality. Three included studies were deemed low-quality due to issues around sampling strategies and a high risk of non-response bias.

### Synthesis of findings

The extracted data were coded and refined into four overarching themes. These were then sub-categorised and organised according to the trial context where relevant. Table 3 outlines the four main themes that developed during the synthesis process with illustrative examples.

**Table 3:** Overarching themes and examples

Overarching themes and their definition	Definition	Example
*Level of acceptability of deferred consent*	Stakeholders’ general level of acceptance towards the use of deferred consent	‘Without disclosure of study outcome, patients and surrogates were glad they were enrolled (90.3%), agreed with exception from informed consent use for their personal enrolment (88.2%), and agreed with the general use of exception from informed consent for the PAMPer study (81.7%)’ [23].
*Research-related factors influencing acceptability of deferred consent*	The effect that trial factors had on stakeholders’ views towards deferred consent; sub-categorised into risks associated with the research; perceived benefit of research; time critical nature of the intervention; levels of emotional stress at the time of trial recruitment	‘The majority (92%) of respondents were willing to be recruited to an emergency research protocol if there were minimal risks involved and 67% if the risks were moderate’ [30].
*Personal characteristics influencing views on deferred consent*	The effect of age, sex, experience, ethnicity, and other patient characteristics on views on deferred consent	‘Older subjects were less likely to offer an agreeable response regarding the use of medications and invasive procedures’ [26].
*Data use after refusal of consent or participant death*	The views of stakeholders regarding the use of participant data upon death or refusal of consent	‘If the relative or patient refused consent, 62% felt the information gathered up to the time of refusal should still be used’ [30].

#### Theme 1:- Level of acceptability of deferred consent

The majority (*n* = 19) of studies reported patients’ and their surrogates’ views towards the acceptability of deferred consent, with four reporting the views of the public and five reporting the views of HCPs. While ten used hypothetical studies to evaluate the acceptability, the remaining studies investigated past experiences with emergency research and deferred consent.

##### Patients and public

Participants were generally accepting of the use of deferred consent [25, 26, 29, 30, 32, 35, 36, 38, 40, 41, 43–45, 47, 49, 50]. However, even in studies reporting positive views, a wide range of acceptability was observed (50–95.6%) [36, 44]. This wide expression of acceptability may be a consequence of the way in which the question was framed to the participants. The three studies which reported negative patient views all investigated patients with acute myocardial infarctions (AMI) or stroke [33, 34, 48]. One was a small US study which interviewed AMI patients using hypothetical scenarios involving deferred consent and found that they were opposed to its use in research investigating procedures but reported greater acceptance for trials investigating approved drugs [33]. The other two studies discussed patients’ actual experiences of emergency research. The high-risk nature of one trial’s intervention (thrombectomy) may have contributed to the low acceptance rates [48]. In the third study, the high risk of recall bias meant that drawing accurate conclusions may not be possible [34]. Studies which reported qualitative data from in-depth interviews provided additional contextual information about acceptability but also found variations in opinion [28, 39, 42, 46].

##### Healthcare professionals

Five studies reported the views of HCPs and researchers, finding largely positive views towards deferred consent which were consistent across the reported countries [24, 27, 31, 40, 42]. The level of research experience may influence HCPs’ and researchers’ views. Interviews with UK research nurses found those with less experience viewed deferred consent as problematic and felt uncomfortable with the process, tending to avoid enrolling patients into trials when prospective written consent was not possible [27]. However, experienced nurses recognised the importance of deferred consent and felt more comfortable dealing with the challenges associated with the process [27]. Deferred consent was viewed as effective, feasible, and ethical by physicians and research coordinators from a tri-national study conducted in Canada, Australia, and New Zealand [31], nearly all of whom (98.2%) had obtained consent from a clinical research participant and on average had over 13 years of experience in their respective professions.

#### Theme 2: Research-related factors influencing acceptability of deferred consent

Factors affecting when deferred consent is considered ethically justified included the risks associated with the research, perceived benefit to the participant, time-critical nature of the intervention, and levels of emotional stress at the time of recruitment.

##### Risk of research

Researchers who used hypothetical scenarios, exploring the effect of the risk of the intervention on the acceptability of deferred consent in particular populations, found a unanimous reduction in acceptability towards deferred consent as risk increased [26, 33, 36, 37, 41, 43, 45, 50]. Patients surveyed in a hospital outpatient department had a 20.1% lower acceptance rate regarding research involving ‘invasive procedures’ than research involving a review of medical records [41]. Twenty-five per cent fewer stroke survivors were willing to be recruited for hypothetical ‘moderate-risk’ research compared to ‘low-risk’ research [26]. In comparison, there was only a 9.9% reduction in surveyed members of the public willing to take part in ‘high-risk’ hypothetical pandemic research than ‘low-risk’ [37].

This effect was also evident in studies investigating interventions of various levels of risk. Acceptance levels towards deferred consent in three low-risk studies (micro-biome, NICE-SUGAR, and PRO-TROPICS) were 73%, 95.6%, and 80.1%, respectively [38, 44, 49]. However, in the higher risk ESCAPE trial, investigating endovascular thrombectomy for acute stroke patients, 78% of participants were opposed to the enrolment process [48].

HCPs suggested the level of risk and study type (observational or interventional) were determinants of how applicable they viewed deferred consent to be, with lower-risk studies being more appropriate and observational studies being more feasible.

##### Perceived benefit of research

Perceived benefit affected the way participants viewed the deferred consent process. Patients enrolled in the PAMPer study (pre-hospital plasma for haemorrhagic shock) were significantly more accepting of RWPC enrolment methods during a hypothetical scenario of reduced mortality compared to scenarios with neutral or negative outcomes [30]. A common misconception by patients was the assumption that their inclusion in research was done in their best interest with doctors giving them ‘the most appropriate treatment’ during clinical trials [29]. In one survey, outpatients inexperienced with medical research believed that ‘whatever the doctors have done, they’ve done for my benefit’; this misconception was also noted in a study of patients after enrolment in pre-hospital resuscitation research [28, 30]. Interestingly, AMI patients, with a greater understanding of research, were considerably opposed to enrolment in procedure-only trials using deferred consent and believed research was inappropriate in emergency situations as the doctor should focus solely on the patients’ interests. The concept of randomisation further highlighted the effect of this misconception as participants’ originally favourable opinions towards deferred consent were considerably reduced when randomisation was made apparent, most likely due to the realisation that they may not receive the most beneficial treatment.

Respondents also acknowledged the importance of benefits for future patients. Altruistic motives for supporting deferred consent were commonly expressed as a precondition for emergency research by patients even when direct benefit to those individuals was unlikely [26, 27, 35, 44]. The need to conduct research in order to advance scientific knowledge was highlighted by several patients who were willing to take part in emergency trials as a result [28, 39, 44]. While patients were more likely to consent to participate in research to help advance medical knowledge, surrogate decision-makers were less likely to support this, focusing more on the medical benefit for their relatives [38, 40]. However, one study found that patient outcomes did not affect the spouses’ views towards enrolment in emergency stroke research [40].

##### Relationship between risk and benefit

Acceptance of deferred consent was associated with a perception that the potential to benefit from research participation outweighed the potential risks [29]. If the condition was severe (e.g. AMI), and proven treatment was available, then participants reported a preference for standard care over experimental research [28], whereas when treatment options were limited, participants acknowledged they would try anything that could help preserve life, supporting a deferred consent approach [28, 50]. A research ethics committee (REC) member in Malawi, where deferred consent is not legally approved, concurred with these views, stating that deferred consent would only be acceptable when the research was potentially life-saving and no current treatments were available [42].

##### Time-critical nature of the intervention

The time-critical nature of an intervention was an influencing factor in accepting deferred consent in several studies [24, 26, 32, 40, 42, 43, 48–50]. Many patients understood that delays to certain treatments could reduce their therapeutic effect or potentially be harmful to them and supported deferred consent as a result [39, 40]. HCPs highlighted the inherent delays that research processes can have on participants receiving the intervention and the impact of the consent model on enrolment. In difficult cases with limited time to approach, assess, consent, and randomise patients prior to treatment provision, research nurses tended to avoid enrolment [27].

##### Impact of the condition and emergency situation on the ability to provide consent

The effect of physical and emotional stress on a patient’s ability to understand trial information during emergency situations was identified as a justification for the use of deferred consent in several studies [39, 46, 49]. The validity of prospective informed consent in these scenarios was questioned by patients who reported being completely unaware of the trial details at the time of signing consent. Women enrolled in a post-partum haemorrhage trial commented that they could have been ‘signing my mortgage away’, and participants were able to recall details about the trial after their involvement [39]. Similar comments were made by stroke and AMI survivors, concluding that deferred consent was appropriate in stressful clinical situations where patients were unable to meaningfully understand the study information [46].

Studies also highlighted how being consulted to participate in research exacerbates an already stressful situation [27, 49]. Sixty-two per cent of patients agreed that it was stressful to be asked about medical research in the ICU and the majority of study respondents preferred the use of deferred consent for this reason [49]. Studies also reported the effect that the patient’s critical condition has on their surrogate, questioning the validity of consent provided by surrogates witnessing distressing situations such as cardiac arrests [24, 40]. For this reason, Honarmand et al. advocated deferred consent as it allows for surrogates to be approached at a time when they may be more able to make an informed decision [38].

#### Theme 3: Personal characteristics influencing views on deferred consent

Inconsistent findings were reported regarding the effect of patient age on the acceptability of deferred consent. While younger members of the Canadian public held more liberal views towards deferred consent [29], age had no effect on patients enrolled in the PAMPer trial [30], and members of the Australian public over the age of 45 were more accepting than younger respondents [50]. Interestingly older stroke survivors were less accepting towards research involving greater risk when interviewed about hypothetical changes to the research they took part in [41].

The effect of respondent ethnicity was also inconsistent. In the ESETT study, evaluating anticonvulsant therapy in patients with status epilepticus, there was no difference in response to general acceptance of enrolment. However, when the lack of prospective consent was emphasised, black participants had lower levels of acceptance [47]. In contrast, a study interviewing AMI and stroke patients concluded that ‘non-white race’ was associated with a preference for not having to sign a consent form [34].

There were some studies which identified that patients with previous ICU or research experience reported more favourable views about deferred consent [37, 44, 45]. However, as previous negative experiences of healthcare were reported to reduce respondents’ acceptability towards emergency research, it is important to acknowledge that two of these studies reported the views of trial survivors, and this positive outcome may have biased their views [28].

Two studies, PRO-TROPICS and NICE-SUGAR, found a significant association between being male and providing consent after research enrolment [38, 44], while the PAMPer trial reported no difference between genders in enrolment rate [30]. Men were reported as being twice as likely to agree to blood sampling for research than women; however, this discrepancy was not apparent in higher risk study scenarios [41].

#### Theme 4: Data use after refusal of consent or death

Some studies using deferred consent have opted to use patient data in circumstances when patients had died prior to regaining capacity and providing informed consent [51–53]. However, views about the process differ [54, 55]. As patients who die during the trial are likely to be the most severely ill, their exclusion introduces selection bias which can affect the validity of the results [38, 42]. HCPs and REC members in Malawi acknowledged the effect of excluding data has on research; however, several questioned the ethics of data use in these circumstances [42]. Most importantly, both studies that reported stakeholder views about data use after death found it was supported in these circumstances [26, 50].

 As well as patient death, the use of data upon declining consent to continue in a study introduces similar issues. Studies reporting a patient preference for the use of collected data up to the point of refusal of continued participation concluded that the majority of patients and surrogates approved of this practice [26, 35, 38].

## Discussion

The findings from this review have provided a greater understanding about stakeholders’ views towards deferred consent which may enable refinements of the consent process in order to achieve a more ethical and effective practice for enrolling incapacitated patients in emergency research. Despite the included studies’ heterogeneity, the narrative synthesis enabled an assessment of the rate of stakeholders’ acceptability towards deferred consent and identified several factors that influenced their views.

The reduction in acceptability of deferred consent as the level of perceived risk increases has also been seen in paediatric emergency medicine, where parental opinion towards deferred consent was positively influenced when informed the research posed no additional risk to their child [7]. Increased risk had a smaller effect on public opinion compared to those of current and former ICU patients which is possibly because they are not able to fully appreciate the circumstances of hypothetical scenarios due to a lack of previous exposure to clinical research [37]. Greater acceptability rates were also observed when participants anticipated that their involvement in research would either benefit themselves or the wider community or they perceived the benefit would outweigh the potential risks of taking part in the research [29]. Although our review excluded paediatric research, parents have similarly reported weighing up the decision as ‘two ends of the scale, the fear of the unknown and the possibility that it might resolve your child’s problem’ [56].

Patients often believed their enrolment in research meant that they would be receiving the most appropriate treatment for their condition. This therapeutic misconception is well described in research ethics literature and could have led to heightened estimations of perceived benefit, undermining the validity of reported acceptability due to an inaccurately perceived risk-benefit ratio [57]. To avoid this, better information should be provided to the public on the basics of clinical trials, and future study participants providing their views on the use of deferred consent must be provided with concise information on the risks and benefits of the study.

Patients and HCPs were accepting the use of deferred consent where delays to treatment initiation could reduce its efficacy and lead to harm [29, 41, 46]. It is important that inclusion in emergency research does not lead to increased time from initial assessment to initiation of the intervention. The inherent delays from research processes such as seeking surrogate consent, could affect patient outcomes and underestimate treatment effects [7]. In these circumstances, researchers have three options: not to enrol critically ill patients unable to provide prospective consent, only enrol patients when surrogates are readily available, or to use alternative consent methods such as deferred consent [58]. The latter is the only option that does not introduce systematic bias.

Participant characteristics were inconsistently associated with deferred consent acceptability, and conflicting findings from previously published literature support the inability to infer conclusions on this topic [59–61]. While inconsistent findings on the effect of ethnicity were reported, the problematic use of homogeneous ethnic groups such as ‘non-white’ and ‘non-black’ in some studies prevented in-depth analysis of the heterogeneous ethnicities of study populations [34, 41]. In all but one study, most participants were of white ethnicity. The small sample size of participants from black and minority ethnic groups may have contributed to the inconclusive findings. It is also possible that the underreporting of minority ethnic groups, who may hold more conservative views towards deferred consent, resulted in overestimations of acceptability in the included studies [62].

Patients and HCPs were supportive of data being used in the case of patient death or up until the point of declining to continue in a study [26, 35, 38, 50]. While this preserves the validity of trial results by preventing selection bias, ethical questions are raised on whether families should be informed of their relatives’ inclusion after death. Such a situation has the potential to cause unwelcome harm to grieving family members. This harm must be weighed up against the possibility that family members may eventually discover that their relative was included in the study, potentially resulting in more distressing events that may receive negative media attention and jeopardise the trial [61].

This review found that HCPs with greater research experience were more willing to enrol patients using deferred consent and held more positive views towards the process [27, 31]. It is notable that paediatric practitioners with no experience have also reported negative views on deferred consent whereas experienced practitioners described how it had improved recruitment rates and the decision-making capacity of patients consenting for their children in research [63].

### Limitations

This systematic review has several limitations. Firstly, the study question relied on a complex search strategy to include the various synonyms used to describe the process of deferred consent. Secondly, while the use of narrative synthesis allowed assessment of the included studies, heterogeneous outcome measures meant that variations in study results could not be reliably attributed to the different trial characteristics. We recognise that many patterns we drew out in our data were only supported by a small number of studies or a small number of participants. Thirdly, as only studies published in the English language were included, stakeholders’ views in other countries may be different to those included in this review. In addition, as previously reported, the findings highlight the importance of how questions in surveys are framed and phrased in relation to the acceptability of deferred consent and the need for caution when interpreting data in this complex area [47].

## Conclusion

This systematic review indicates that the use of deferred consent would be most acceptable to stakeholders during low-risk emergency research in incapacitated patients with critical conditions if the treatment has a narrow therapeutic window and there is potential for patients to benefit from their inclusion. The results from this review could be used to design guidance for RECs to use while reviewing the use of deferred consent in proposed research studies as well as a framework for the conduct of deferred consent in clinical research practice. Future research should aim to develop and evaluate such guidance. Future studies should also concentrate on the opinions of HCPs and researchers whose views have not been explored in as much depth as patients and members of the public.

## Supplementary Information

**Additional file 1.** PRISMA checklist.

**Additional file 2.** MEDLINE search strategy.

**Additional file 3.** Systematic Review data extraction form.

**Additional file 4.** MMAT checklist.

## Data Availability

The datasets used and/or analysed during the current study are available from the corresponding author on reasonable request.

## Abbreviations

RWPC: Research without prior consent
REC: Research Ethics Committee
EU: European Union
HCP: Healthcare professional
AMI: Acute myocardial infarction
