# Supplementary material for: Trials using deferred consent in the emergency setting: a systematic review and narrative synthesis of stakeholders’ attitudes
Source: Trials. 2022 May 16;23:411. doi: 10.1186/s13063-022-06304-x (PMC9109432; doi:10.1186/s13063-022-06304-x)
Supplement: Supplementary file 2 — Additional file 2. MEDLINE search strategy. [file 13063_2022_6304_MOESM2_ESM.docx]

**Supplementary file 2: MEDLINE search strategy**

| 1 | exp Informed Consent/ |
| --- | --- |
| 2 | ((consent* or decision mak*) adj3 (inform* or waive* or defer* or model* or presume* or procedure* or method* or surrogate* or communit* or universal or delay* or retrospective* or exception* or alternative)).mp. [mp=title, abstract, original title, name of substance word, subject heading word, floating sub-heading word, keyword heading word, organism supplementary concept word, protocol supplementary concept word, rare disease supplementary concept word, unique identifier, synonyms] |
| 3 | 1 or 2 |
| 4 | (emergen* or urgen* or (care* adj3 (critical or intensive)) or paramedic* or ambulanc* or cardiac arrest* or myocardial infarction* or obstetric* or stroke* or trauma*).mp. [mp=title, abstract, original title, name of substance word, subject heading word, floating sub-heading word, keyword heading word, organism supplementary concept word, protocol supplementary concept word, rare disease supplementary concept word, unique identifier, synonyms] |
| 5 | exp Emergency Service, Hospital/ |
| 6 | exp Critical Care/ |
| 7 | exp Ambulances/ |
| 8 | exp Heart Arrest/ |
| 9 | exp Stroke/ |
| 10 | 4 or 5 or 6 or 7 or 8 or 9 |
| 11 | 3 and 10 |
| 12 | ((health* or medic* or care*) adj3 (profession* or work* or provider* or staff or practitioner* or worker*)).mp. [mp=title, abstract, original title, name of substance word, subject heading word, floating sub-heading word, keyword heading word, organism supplementary concept word, protocol supplementary concept word, rare disease supplementary concept word, unique identifier, synonyms] |
| 13 | exp Health Personnel/ |
| 14 | nurs*.mp. [mp=title, abstract, original title, name of substance word, subject heading word, floating sub-heading word, keyword heading word, organism supplementary concept word, protocol supplementary concept word, rare disease supplementary concept word, unique identifier, synonyms] |
| 15 | physician*.mp. [mp=title, abstract, original title, name of substance word, subject heading word, floating sub-heading word, keyword heading word, organism supplementary concept word, protocol supplementary concept word, rare disease supplementary concept word, unique identifier, synonyms] |
| 16 | doctor*.mp. [mp=title, abstract, original title, name of substance word, subject heading word, floating sub-heading word, keyword heading word, organism supplementary concept word, protocol supplementary concept word, rare disease supplementary concept word, unique identifier, synonyms] |
| 17 | exp Patients/ |
| 18 | patient*.mp. [mp=title, abstract, original title, name of substance word, subject heading word, floating sub-heading word, keyword heading word, organism supplementary concept word, protocol supplementary concept word, rare disease supplementary concept word, unique identifier, synonyms] |
| 19 | public.mp. [mp=title, abstract, original title, name of substance word, subject heading word, floating sub-heading word, keyword heading word, organism supplementary concept word, protocol supplementary concept word, rare disease supplementary concept word, unique identifier, synonyms] |
| 20 | exp Family/ |
| 21 | (famil* adj3 member*).mp. [mp=title, abstract, original title, name of substance word, subject heading word, floating sub-heading word, keyword heading word, organism supplementary concept word, protocol supplementary concept word, rare disease supplementary concept word, unique identifier, synonyms] |
| 22 | (relative or relatives).mp. [mp=title, abstract, original title, name of substance word, subject heading word, floating sub-heading word, keyword heading word, organism supplementary concept word, protocol supplementary concept word, rare disease supplementary concept word, unique identifier, synonyms] |
| 23 | participant*.mp. [mp=title, abstract, original title, name of substance word, subject heading word, floating sub-heading word, keyword heading word, organism supplementary concept word, protocol supplementary concept word, rare disease supplementary concept word, unique identifier, synonyms] |
| 24 | carer.mp. [mp=title, abstract, original title, name of substance word, subject heading word, floating sub-heading word, keyword heading word, organism supplementary concept word, protocol supplementary concept word, rare disease supplementary concept word, unique identifier, synonyms] |
| 25 | third-party.mp. [mp=title, abstract, original title, name of substance word, subject heading word, floating sub-heading word, keyword heading word, organism supplementary concept word, protocol supplementary concept word, rare disease supplementary concept word, unique identifier, synonyms] |
| 26 | surrogate.mp. [mp=title, abstract, original title, name of substance word, subject heading word, floating sub-heading word, keyword heading word, organism supplementary concept word, protocol supplementary concept word, rare disease supplementary concept word, unique identifier, synonyms] |
| 27 | consultee.mp. [mp=title, abstract, original title, name of substance word, subject heading word, floating sub-heading word, keyword heading word, organism supplementary concept word, protocol supplementary concept word, rare disease supplementary concept word, unique identifier, synonyms] |
| 28 | exp Proxy/ |
| 29 | proxy.mp. [mp=title, abstract, original title, name of substance word, subject heading word, floating sub-heading word, keyword heading word, organism supplementary concept word, protocol supplementary concept word, rare disease supplementary concept word, unique identifier, synonyms] |
| 30 | representative.mp. [mp=title, abstract, original title, name of substance word, subject heading word, floating sub-heading word, keyword heading word, organism supplementary concept word, protocol supplementary concept word, rare disease supplementary concept word, unique identifier, synonyms] |
| 31 | 12 or 13 or 14 or 15 or 16 or 17 or 18 or 19 or 20 or 21 or 22 or 23 or 24 or 25 or 26 or 27 or 28 or 29 or 30 |
| 32 | 11 and 31 |
| 33 | (perception* or perceive* or attitude* or view* or encounter* or experience* or description* or opinion* or need* or concern* or belie*).mp. [mp=title, abstract, original title, name of substance word, subject heading word, floating sub-heading word, keyword heading word, organism supplementary concept word, protocol supplementary concept word, rare disease supplementary concept word, unique identifier, synonyms] |
| 34 | exp Attitude/ |
| 35 | exp Perception/ |
| 36 | 33 or 34 or 35 |
| 37 | 32 and 36 |
| 38 | exp Adult/ |
| 39 | 37 and 38 |
| 40 | limit 39 to (English language and yr="1996 -Current") |
