# Supplementary material for: Trials using deferred consent in the emergency setting: a systematic review and narrative synthesis of stakeholders’ attitudes
Source: Trials. 2022 May 16;23:411. doi: 10.1186/s13063-022-06304-x (PMC9109432; doi:10.1186/s13063-022-06304-x)
Supplement: Supplementary file 3 — Additional file 3. Systematic Review data extraction form. [file 13063_2022_6304_MOESM3_ESM.docx]

**Supplementary file 3: Systematic Review data extraction form**

| Date of Review: | Reviewer: |
| --- | --- |
| Study ID: | Journal: |
| Year of Publication: | First Author: |
| Article title: |  |

| STUDY DETAILS |  |
| --- | --- |
| Sources of funding |  |
| Study design |  |
| Authors’ objective(s) |  |
| METHODS |  |
| Population: |  |
| Setting: |  |
| Country: |  |
| Inclusion/ exclusion criteria: |  |
| Method of recruitment |  |
| Method of data collection |  |
| Method of data analysis |  |
| Research context |  |
| Consent model |  |
| Phenomena of interest |  |
| Measures |  |
| Definition of outcomes |  |
| Duration of study |  |
| RESULTS |  |
| Total number of participants by type |  |
| Participants’ characteristics: |  |
| Findings/ theme 1 |  |
| Findings/ theme 2 |  |
| Findings / theme 3 |  |
| Findings / theme 4 |  |
| Findings / theme 5 |  |
| Findings / theme 6 |  |
| Findings / theme 7 |  |
| Authors’ comments on strengths/ weaknesses / limitations |  |
| SUMMARY |  |
| Authors’ overall conclusions: |  |
